# Cystic Echinococcosis: A Case of Extrahepatic Intra-Abdominal Involvement

**DOI:** 10.1155/2017/8919546

**Published:** 2017-01-17

**Authors:** Patrícia Leitão, André Carvalho, Teresa Fernandes, José Gonçalves

**Affiliations:** Radiology Department, Centro Hospitalar São João, Porto, Portugal

## Abstract

Hydatid disease, or echinococcal disease, is a parasitic infestation caused by the larval stage of the* Echinococcus* tapeworm and it primarily affects the liver and lung but involvement of other organs is also possible secondary to peritoneal seeding or hematogeneous dissemination. We describe a rare case of extensive abdominal disease, with lesions affecting the liver, peritoneum, and lesser omentum, requiring aggressive surgical intervention. Complementary diagnostic exams were crucial to reach the diagnosis and evaluate the extension of the disease.

## 1. Case Report

We report a case of a 49-year-old Caucasian man who presents to our attention with a history of long term fatigue, increased abdominal girth, and right upper quadrant pain. The past medical history is unremarkable, with mild mental impairment secondary to neonatal meningitis and arterial hypertension; he has no past surgical history, has always lived in an apartment in a semirural region in Portugal, has never traveled outside his country, and denies contact with animals except for dogs during the childhood. Familiar history is irrelevant and no other family members had the same complaints.

The symptoms started in 2004 and at that time he performed general blood tests (without abnormalities except for elevated hepatic enzymes) and an ultrasound that revealed multiple hepatic complicated cysts, suggestive of hydatid cysts (HC). The patient was prescribed albendazole 400 mg bid, which he had taken for one year. Posterior ultrasound showed persistence of the cysts, so it was decided to perform laparoscopic cystectomy. In 2013, the patient seeks again for medical attention with symptoms of asthenia, abdominal generalized pain, postprandial fullness, and vomiting.

On admission to our facilities he was conscient, collaborative and fully oriented, and apyretic and the vital signs were within the normal range. Physical examination revealed hydrated mucosa and no signs of jaundice, but abdominal distention and diffuse abdominal tenderness. Complementary diagnostic exams (blood tests, ultrasound, and CT) were repeated.

## 2. Methods

### 2.1. Blood Tests

The routine blood tests are not specific and varies according to the organs involved. In the case of liver involvement there may be elevation of liver enzymes and if rupture to the biliary tree occurs, bilirubin and alkaline phosphatase levels may rise. Other possible findings are leukocytosis (if there is infection of the cyst), eosinophilia (occurring in only 25% of the patients), and hypogammaglobulinemia (present in 30% of the cases) [1].

Serologic tests available are numerous and they have a role not only in the diagnosis but also in evidencing recurrence. However, their sensitivity and specificity are not optimal, with positive tests occurring in only 54.8% of cases with early or inactive cysts [2]. Detectable antibodies depend on cysts location, cysts integrity, and vitality of the larval cyst; the other problem is cross-reactions, especially with other parasitic diseases. A way to overcome these limitations is to combine several tests [2] and the most used approach is initial screening with latex agglutination, double diffusion, indirect hemagglutination, and enzyme-linked immunosorbent assay, followed by confirmatory tests using specific antigens, for example, the antigen 5 immunoelectrophoresis and immunoblotting [3].

### 2.2. Thoracic, Abdominal, and Pelvic Computed Tomography (CT)

On CT, cysts exhibit water attenuation and thin, nondiscernible walls. In the case of a hydatid cyst, it typically demonstrates a high-attenuation wall at unenhanced CT even if there is no calcifications [4]. Other findings are the detachment of the laminated membrane from the pericyst (the outer layer of the cyst), identified as linear areas of increased attenuation within the cyst, and daughter cysts, round structures in the periphery, with lower attenuation compared to that of the mother cyst [4, 5].

HCs are classified into four types on the basis of their imaging appearance, with type I being simple cysts, type II being cysts with daughter cysts within them and/or floating membranes or vesicles (type II can be divided into IIa: round daughter cysts at the periphery; IIb: larger, irregularly shaped daughter cysts that occupy almost the entire volume of the mother cyst; and IIc: cysts with scattered calcifications and occasional daughter cysts), type III being total calcified cyst (dead cysts), and type IV being complicated cysts (with rupture and superinfection) [5].

### 2.3. Abdominal and Pelvic Magnetic Resonance Imaging (MRI)

In general cysts appear on MR imaging as homogenous low-signal intensity lesions in T1-weighted and homogenous high signal intensity in T2-weighted sequences. On the other hand, hydatid cysts may have a low-signal-intensity rim on T2-weighted sequences because of the presence of the pericyst. Daughter cysts are hypointense relative to the intracystic fluid (in agreement with CT findings) on T1-weighted images and hyperintense on T2-weighted images. Irregularities of the cyst rim seen on MRI reflect detachment of the membranes [5–7].

Although there is no current consensus on the use of diffusion-weighted imaging (DWI) and apparent diffusion coefficients (ADC) ratios for the differentiation between simple cysts and type 1 hydatid cysts, some recent reports advocate their use, showing that hydatid cysts have hyperintense signal in DWI and lower ADC values due to their content rich in proteins, glucose, and scolices [6, 7].

In conjunction with CT, MRI plays a key role in recognizing cyst complications, such us rupture and infection.

## 3. Results

**Figure 1:**
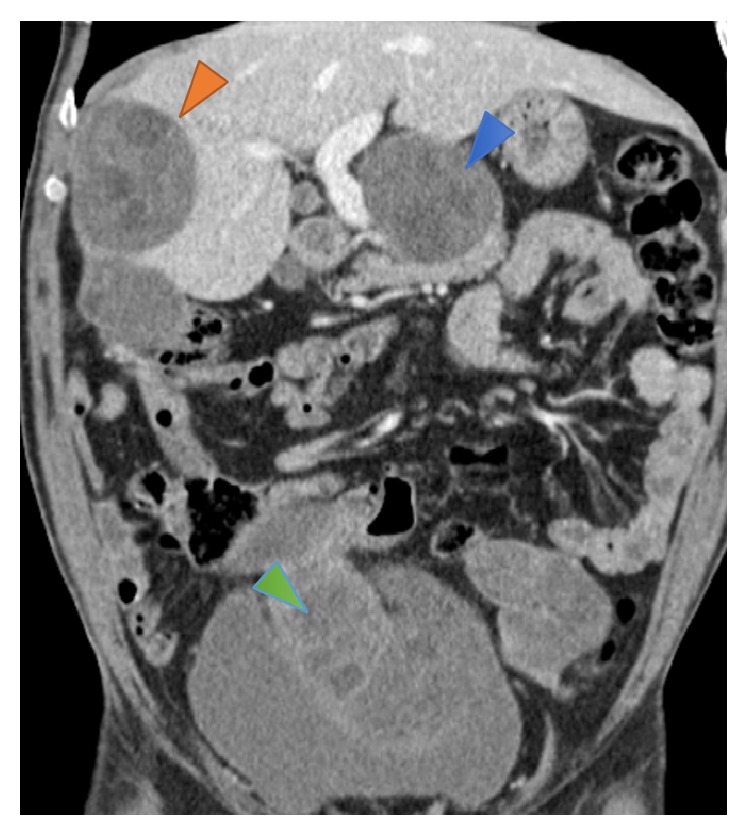
Coronal contrast enhanced CT image shows multiple abdominal hypodense lesions within liver (orange arrowhead) and lesser omentum (blue arrowhead) and peritoneum and above the bladder (green arrowhead).

**Figure 2:**
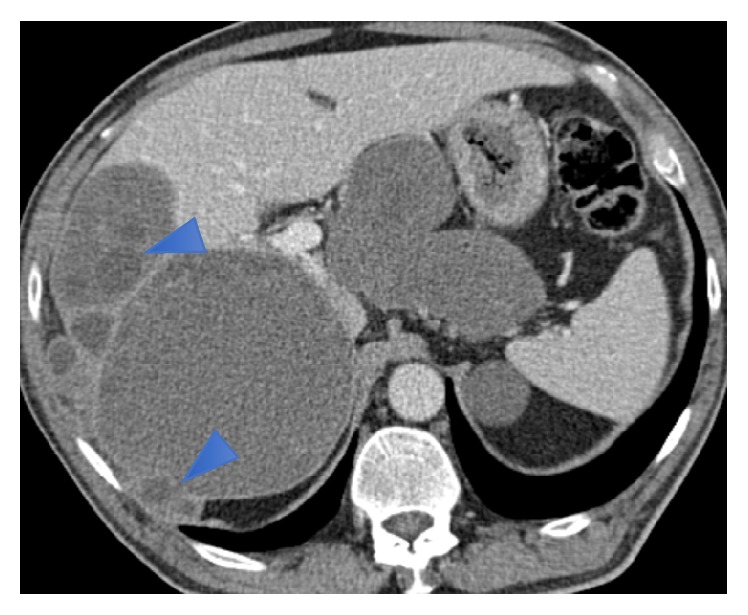
Axial contrast enhanced CT image nicely shows the hypodense lesions with water density and multiple smaller cysts at the periphery of the larger cysts (daughter cysts, arrowheads).

Abdominal and pelvic CT confirmed recurrent disease, with large cysts located in the right hepatic lobe, peritoneal cavity, and kidneys (Figure 1). The lesions seen in the liver and peritoneum were well defined, well capsulated, fluid density lesions, some with peripheral calcifications; the presence of unilocular cysts with multiple peripheral daughter cysts within them (hydatid cysts type IIa) confirmed the diagnosis of hydatidosis (Figure 2). The cysts located in the kidneys had not the typical characteristics of hydatidosis, so it was not possible to confirm the infestation of these organs with this imaging technique. There were also hydatid cysts close to the superior portion of the pancreas, but they were apparently in the lesser omentum and not involving the pancreas. Thoracic CT revealed no significant alterations.

**Figure 3:**
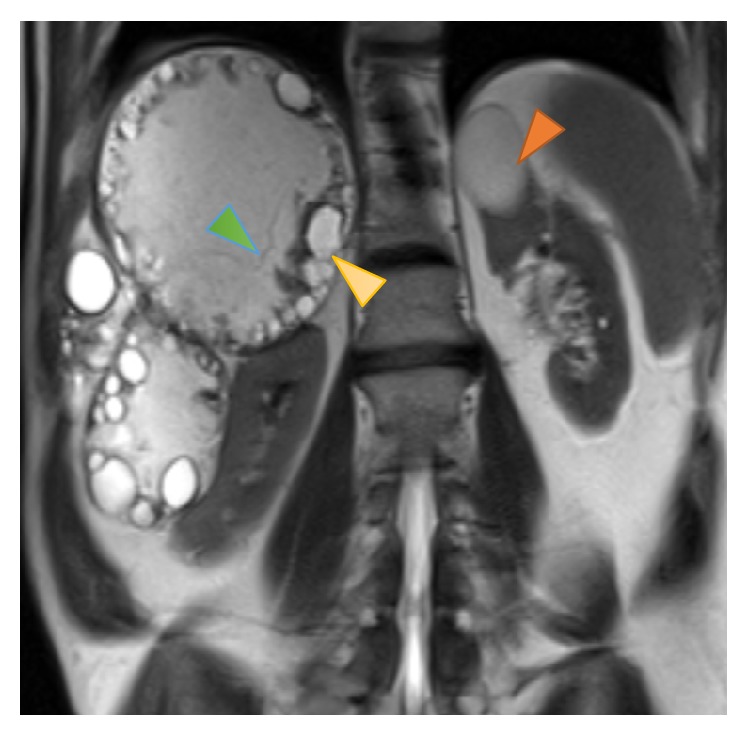
Coronal T2-weighted MR image shows multiple abdominal hyperintense lesions with smaller peripheral cysts compatible with daughter cysts (yellow arrow) and also thin linear septation, compatible with the detachment of the laminated membrane of the pericyst (green arrowhead). Note also a simple renal cyst (orange arrowhead), probably an incidental finding.

**Figure 4:**
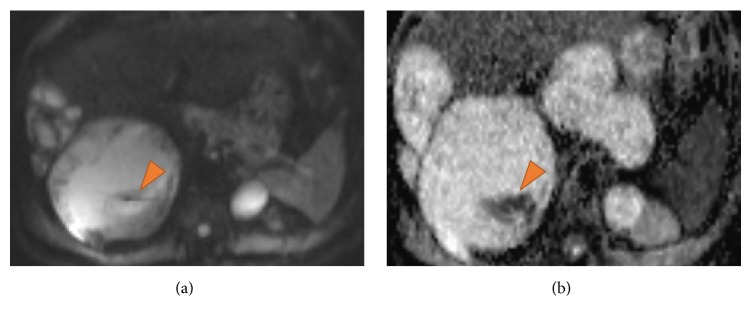
(a) DWI *b* = 50 and (b) ADC mapping at the levels of the liver hydatid cysts and renal cyst show higher signal intensity of the renal cyst at DWI, suggesting a simple cyst. There is also an area of water motion restriction identified as a focus of high signal intensity at DWI and low signal on the ADC map (arrows).

Although the CT findings were characteristic of hydatidosis and no further confirmation was needed, abdominal MRI was also performed to help in differentiating renal simple cysts from hydatid cysts. MR imaging confirmed the diagnosis showing the hydatid cysts with daughter cysts associated (Figure 3) and DWI sequence and ADC ratios favored the presence of simple cysts in kidneys (Figure 4).

Blood tests showed normal white and red cells count, mild elevation of transaminases with normal cholestatic enzymes values, normal sedimentation rate, and normal renal function. Serologic tests showed elevated anti-*Echinococcus* antibodies (Immunoglobulin G).

Because of the extension of the disease it was decided to repeat three months of albendazole and then perform exploratory laparotomy with partial hepatectomy and total pericystectomy of the peritoneal cysts.

## 4. Discussion

This case report describes the clinical presentation of extensive and recurrent abdominal hydatidosis. The main differential diagnosis of hydatid disease is simple cysts, cystic metastasis, pancreatic pseudocysts, and cystic teratoma; in the case of complicated or calcified cysts (type III or IV), the differentials are abdominal abscesses, tuberculosis, and hepatocellular carcinoma [1, 4].

Hydatid disease is a common parasitic zoonosis and its mortality, morbidity, and socioeconomic burden makes it a significant public health problem. Although more prevalent in developing countries, HD is a worldwide zoonosis, being reported in over 100 countries [4].

There are three forms of echinococcosis affecting humans and caused by the larval stage (metacestodes) of the* Echinococcus *tapeworm:* E. granulosus* (responsible for cystic echinococcosis, the most common type of hydatid disease),* Echinococcus multilocularis* (causing alveolar echinococcosis), and* Echinococcus vogeli* and* Echinococcus oligarthus* (causing polycystic echinococcosis) [8, 9].

Dog and other canids are the definite host and human infestation occurs with ingestion of the parasite eggs from infected animals (normally cattle). The disease is characterized by slowing growing cysts, more commonly in liver >65% of the cases [10], although they can affect almost every organ in the body.

The clinical features depend on the affected organs but are usually related to compression symptoms; besides these, there are many potential complications, namely, local complications (superinfection and rupture) or secondary involvement of other organs due to hematogenous dissemination. Rupture can occur spontaneously, during fine needle biopsy or excision surgery, and can evoke mild to serious allergic reactions or secondary recurrence of the disease.

The diagnosis is based on clinical history, physical examination, and imaging findings, the latter playing a key role in diagnosing and staging of the disease.

There are various forms of treatment of hydatidosis and the best approach depends on the cyst type, location, and comorbidities of the patient [11]. Small, calcified cysts do not require treatment but should be monitored [11, 12]. Treatment options can be divided into chemotherapy (benzimidazolic drugs) and surgery, which consist of PAIR (Puncture, Aspiration, Injection, Reaspiration), PPDC (Percutaneous Puncture with Drainage and Curettage), conservative surgery (open cystectomy with or without omentoplasty), and radical surgery (total pericystectomy or partial hepatectomy). Palliative treatment consists of simple tube drainage of infected cysts or communicating cysts.

Anthelmintics can be used as single treatment or as neoadjuvant treatment; the cure rate with medical treatment alone is only 30% [13] so this is an option just in cases of inoperable disease or high surgical risk.

PAIR is the technique of choice for type I cysts with five or more centimeters of diameter, type II cysts, and infected cysts, if they are accessible for puncture [14]. Conservative and radical surgeries are the preferred methods in patients after recurrence and in extensive disease, if patient has low surgical risk [15].

In summary, hydatid disease remains a significant medical problem, especially in endemic countries. Although liver and lung are the most commonly involved organs, in 10% of cases it occurs in other locations. The overall incidence of peritoneal cavity involvement is 13% [16] and it can be divided into primary involvement (only 0.5–5% of peritoneal cases [17]) or secondary to hepatic cysts surgery or spontaneous/traumatic rupture (the most common). In this case, the high clinical suspicion based on the symptoms and their duration and the knowledge of the epidemiological data, complemented by typical imaging findings, helped to reach the diagnosis.
